# Gender specific decrease of a set of circulating N-acylphosphatidyl ethanolamines (NAPEs) in the plasma of Parkinson’s disease patients

**DOI:** 10.1007/s11306-019-1536-z

**Published:** 2019-05-03

**Authors:** Zeeshan Hamid, Abdul Basit, Silvia Pontis, Fabrizio Piras, Francesca Assogna, Paola Bossù, Francesco Ernesto Pontieri, Alessandro Stefani, Gianfranco Spalletta, Pietro Franceschi, Angelo Reggiani, Andrea Armirotti

**Affiliations:** 1 D3Validation, Fondazione Istituto Italiano di Tecnologia, via Morego 30, 16163 Genoa, Italy; 2 Scuola Superiore Sant’Anna, via Piazza Martiri della Libertà, 33, 56127 Pisa, Italy; 3 Laboratorio di Neuropsichiatria, IRCCS Fondazione Santa Lucia, Via Ardeatina, 306, 00179 Rome, Italy; 4 Department of Neuroscience, Mental Health and Sensory Organs (NESMOS), Sapienza University, Via di Grottarossa 1035, 00189 Rome, Italy; 5 Department of Medicine of Systems, Tor Vergata University, Viale Oxford 81, 00133 Rome, Italy; 6 Computational Biology Unit, Research and Innovation Centre, Fondazione Edmund Mach, Via E. Mach 1, San Michele all’Adige, TN Italy; 7 Analytical Chemistry Lab, Fondazione Istituto Italiano di Tecnologia, via Morego 30, 16163 Genoa, Italy

**Keywords:** Parkinson’s disease, Lipid biomarkers, NAPE

## Abstract

**Introduction:**

Current markers of Parkinson’s disease (PD) fail to detect the early progression of disease state. Conversely, current omics techniques allow the investigation of hundreds of molecules potentially altered by disease conditions. Based on evidence previously collected by our group in a mouse model of PD, we speculated that a particular set of circulating lipids might be significantly altered by the pathology.

**Objectives:**

The aim of current study was to evaluate the potential of a particular set of N-acyl-phosphatidylethanolamines (NAPEs) as potential non-invasive plasma markers of ongoing neurodegeneration from Parkinson’s disease in human subjects.

**Methods:**

A panel of seven NAPEs were quantified by LC–MS/MS in the plasma of 587 individuals (healthy controls, n = 319; Parkinson’s disease, n = 268); Random Forest classification and statistical modeling was applied to compare Parkinson’s disease versus controls. All p-values obtained in different tests were corrected for multiplicity by controlling the false discovery rate (FDR).

**Results:**

The results indicate that this panel of NAPEs is able to distinguish female PD patients from the corresponding healthy controls. Further to this, the observed downregulation of these NAPEs is in line with the results in plasma of a mouse model of Parkinson’s (6-OHDA).

**Conclusions:**

In the current study we have shown the downregulation of NAPEs in plasma of PD patients and we thus speculate that these lipids might serve as candidate biomarkers for PD. We also suggest a molecular mechanism, explaining our findings, which involves gut microbiota.

**Electronic supplementary material:**

The online version of this article (10.1007/s11306-019-1536-z) contains supplementary material, which is available to authorized users.

## Introduction

Parkinson’s disease (PD) is a neurodegenerative disorder that affects motor functions and cognitive/neuropsychiatric performances such as memory, speech, executive functions, praxis, mood, thought and others. With the progressive ageing of the population, the incidence of this condition is constantly increasing, causing a huge challenge for the healthcare systems. Epidemiological data suggest that in subjects above 60 years of age as many as 1% of the worldwide population in the western countries are PD sufferers, with a rising prevalence associated to age. Moreover, PD is causing more than 100,000 deaths a year worldwide (Shulman et al. 2011). At present, PD can only be diagnosed when clinical symptoms are already detectable and thus proper medical treatment can only start when the disease is established, with a strong reduction in the efficiency of the therapeutic intervention. No validated PD molecular biomarkers are currently available for the clinical practice, although many have been proposed (Bolner et al. 2016; Gabriel et al. 2017; Pan et al. 2014; Peran et al. 2010). From this perspective, it would be highly beneficial to identify circulating molecules, easy to measure with a simple blood test and highly correlated with progression and severity of neurodegeneration, which could serve as an early marker of the onset of disease. Given the role of lipids, particularly membrane lipids, in initiating the aggregation of alpha-synuclein, which is considered to be the initial trigger for development of Parkinson’s disease (Ruiperez et al. 2010), recent literature supports the hypothesis that lipids in general might play a role in the development of the disease, particularly at its earlier stages. Some time ago, while testing a new setup for untargeted lipidomics, we found that in a mouse model of PD, i.e. the striatal injection of 6 hydroxy-dopamine (6-OHDA), there is a strong upregulation of seven polyunsaturated phospholipids in the dorsal striatum as early as 48 h from disease induction (Basit et al. 2016). These lipids are N-acyl phosphatidylethanolamines, bearing either docohexaenoic (22:6) or arachidonic (20:4) acid residues in Sn2 position of the glycerol moiety and a stearic (18:0) or palmitic (16:0) acid acyl chain linked to ethanolamine (Coulon et al. 2012). Since these lipids can relatively easily be measured in plasma, we expanded the study by quantifying the same set of lipids in plasma of 6-OHDA animals, at the same early stage timepoint (48 h), Finally, given the potential of our findings in the context of PD diagnosis, we conducted an exploratory study, by measuring the levels of these lipids in the plasma of a group of human subjects diagnosed with PD.

## Methods

### Animal experiments

Male 8–10 weeks old mice were anesthetized with a mixture of ketamine/xylazine (100 and 10 mg/kg body weight, respectively) and placed in a stereotaxic frame with a mouse-adaptor (Stoelting, Wood Dale, USA). 6-hydroxy-DA was dissolved at a concentration of 3.2 μg/μL of ice-cold 0.9% saline solution containing 0.02% ascorbate. Two injections of 1 μL each were made at the following brain atlas coordinates (in mm relative to bregma and dural surface, Paxinos and Franklin 2001): (i) AP = + 1.0, L = − 2.1, DV = − 2.9; and (ii) AP = + 0.3, L = − 2.3, DV = − 2.9. Sham lesions were carried out by 1 μL injection of 0.02% ascorbic acid-saline at the same coordinates. Forty-eight hours after 6-hydroxy-DA injection, mice were anesthetized with chloral hydrate (450 mg/kg) and killed by decapitation; the blood was collected and plasma was separated and stored at − 80 °C until analysis. (Basit et al. 2016). All procedures were performed in compliance with Italian regulations on protection of animals used for experimental and other scientific purposes (D.M. 116192) as well as with European Economic Community regulations (O.J. of E.C. L 358/1 12/18/1986). The study was approved by the Italian Ministry of Health with code n° 095 for Istituto Italiano di Tecnologia, Animal Facility.

### Recruitment of human subjects

PD participants were assessed at the Laboratory of Neuropsychiatry of the I.R.C.C.S. Santa Lucia Foundation in Rome. Inclusion criteria for PD patients were: (i) diagnosis of idiopathic PD according to the international guidelines (Folstein et al. 1975) (ii) Mini Mental State Examination (MMSE) score > 26 (Emre et al. 2007) (iii) no dementia according to the Movement Disorder Society clinical diagnostic criteria (Iorio et al. 2013) using an extensive neuropsychological battery. Exclusion criteria were (1) presence of major non stabilized medical illnesses (i.e., nonstabilized diabetes, obstructive pulmonary disease or asthma, hematologic/oncologic disorders, vitamin B12 or folate deficiency, pernicious anemia, clinically significant and unstable active gastrointestinal, renal, hepatic, endocrine or cardiovascular disorders and recently treated hypothyroidism. (2) known or suspected history of alcoholism, drug dependence and abuse, head trauma, and mental disorders (apart from mood or anxiety disorders) according to the DSM-IV TR criteria [American Psychiatric Association (2000): Diagnostic and Statistical Manual of Mental Disorders, 4th ed. Washington, DC: Task Force. (3) history of neurological diseases other than idiopathic PD. (4) unclear history of chronic dopaminergic treatment responsiveness. (5) MRI scans lacking signs of focal lesions as computed according to the semi-automated method recently published by our group (McKhann et al. 2011) (minimal diffuse changes or minimal lacunar lesions of white matter (WM) were, however, allowed). All included PD patients were under stable dopaminergic therapy and were at stage I or II of the disease. Furthermore, they were not receiving deep brain stimulation and were not under continuous dopaminergic stimulation by subcutaneous apomorphine or intrajejunal L-Dopa. Inclusion criteria for control subjects were: (i) vision and hearing sufficient for compliance with testing procedures; (ii) laboratory values within the appropriate normal reference intervals; and (iii) neuropsychological domain scores above the cutoff scores, corrected for age and educational level, identifying normal cognitive level in the Italian population. Exclusion criteria were (i) dementia diagnosis, according with DSM-IV criteria or MCI according with Petersen criteria, and confirmed by a comprehensive neuropsychological battery and (ii) MMSE score < 26 according with standardized norms for the Italian population (Dellasega and Morris 1993). At recruitment, patients fulfilled an anamnestic interview including information about major medical illnesses (including neurological conditions other than PD), substance abuse, presence of primary psychiatric disorders, dopaminergic treatment responsiveness, presence of cerebrovascular disease. Patients were also screened for the presence of dementia. After blood sampling, patients’ identity was masked by a random code and all personal information were deleted. The study was approved and financially supported by the Italian Ministry of Health (Grant RF-2013-02359074). All the participants or related caretakers gave their written consent to the enrollment. The present study was approved by the Ethical Committee of IRCCS Santa Lucia Foundation (CE-AG4-Prog.149).

### Human blood sample collection

Blood samples (10 ml) from all participants were, after overnight fasting (11 ± 1 h), collected from forearm veins in BD Vacutainer tubes containing ethylenediaminetetracetic acid (Beckton Dickinson, Franklin Lakes, NJ). Plasma aliquots were prepared by centrifugation at 3000 rpm at 4 °C for 15 min and stored at − 80 °C until analysis.

### NAPE quantification

NAPEs were extracted from 0.1 ml of plasma samples with 1 ml of ethanol containing the synthetic exogenous 18:0-22:6-N17:0 NAPE as an internal standard. The solution was vortexed for 1 min then centrifuged at 3500 g at 4 °C for 10 min. The supernatant was then collected and dried under nitrogen stream. The dried samples were then resuspended in 0.1 ml of 9:1 MeOH/CHCl_3_ solution for LC–MS/MS analysis. Targeted analysis of the samples was then carried out on an Acquity UPLC system coupled with a Xevo TQ-MS triple quadrupole mass spectrometer. Full details of the analytical method are given in the Supplementary Data file. The samples were analyzed over a broad time span (December 2015 to March 2017) in a randomized order, acquiring the same number of controls and disease subjects in each analytical session. Despite the high reproducibility of the analytical method, some residual batch effect was visible in the overall dataset. To compensate for that, a specific batch removal strategy was implemented and it is discussed in the Supplementary Method file.

### Statistics

All statistical analyses were performed in R (https://www.R-project.org/). Welch t-test was applied to statistically compare 6-OHDA versus sham mice. The concentration of the metabolites were log transformed to correct for the heteroscedasticity typical of metabolomics data (van den Berg et al. 2006). A generalized linear modeling (GLM) approach (Venables and Ripley 2002) was used to compare PD patients versus control subjects. In order to relate the intercept of the model to the average concentration of the lipids in the samples, the variable ‘age’ was mean centered and scaled (Gelman and Hill 2007). All p values obtained in the different tests were corrected for multiplicity by applying a False Discovery Rate (FDR) (Franceschi et al. 2013). Multivariate data analysis was performed by applying Random Forests classification separately to male and female subjects. A classification tree based approach was selected because the importance of the individual variables (the individual NAPEs and the Age) can be naturally inspected at the end of the model optimization phase. To avoid overfitting, the following validation scheme was used. (1) the overall dataset was repeatedly (100 times) split in training and test subsets (0.8, 0.2) and separate Random Forests models were fit on the data. (2) The performance of each model was assessed in terms of the AUC of the ROC curve built on the test sample. (3) As an additional validation step, the same scheme was applied to 100 random permutation of the sample class labels. The classifier parameter ‘mtry’ (Number of variables randomly sampled as candidates at each split) was optimized by five-fold repeated cross validation (3 times) by using the caret R package. As in many tree-based approaches, the variable importance in Random Forests was assessed by considering how much a single variable is effective in splitting the data in “pure” classes. The purity of the classes was measured in terms of the Gini impurity index (Wehrens 2011). The index considers values in the [0,1] interval: lower values are associated with higher levels of purity. Important variables are then those able to produce the strongest decrease in the Gini index when “included” in the model. Since Random Forests relies on the construction of a large number of trees, the decrease in Gini is averaged across all the trees which are including a specific variable, so that the variable importance is assessed in terms of Mean Decrease of Gini index. In our validation scheme, each one of the training-test splits was producing an independent model with its own variable importance measure. The individual values were finally combined calculating the medians of each variable importance and estimating the variability of this importance in terms of the 5 and 95 percentiles of the empirical distribution of mean decrease in Gini index. Random Forest classification analysis (Breiman 2001) was performed by using R Random Forest package.

## Results and discussion

**Fig. 1:**
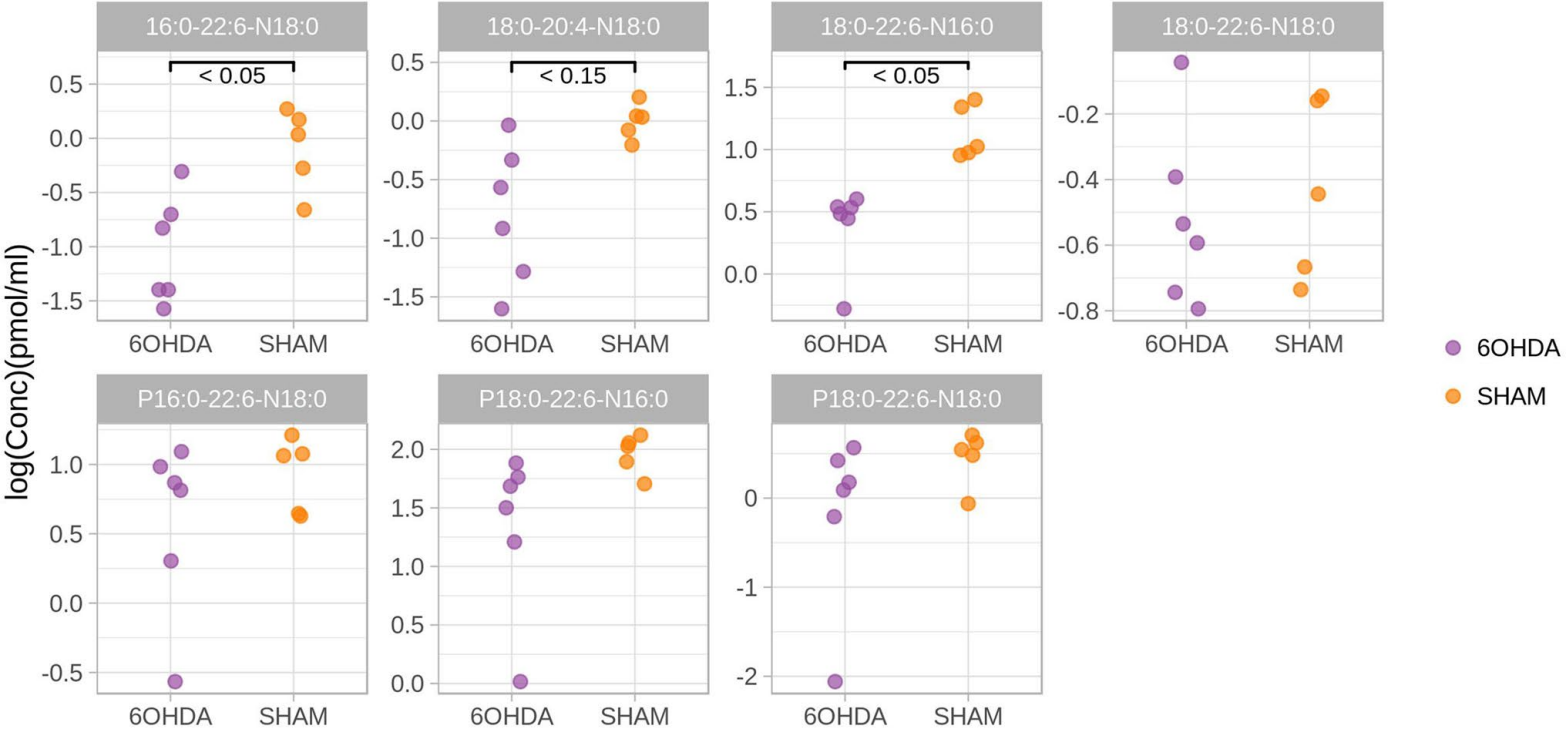
Results of a 2-tails t-test (with FDR correction for multiple testing) comparing the levels of seven NAPEs in the plasma of male mice treated with 6-OHDA, sampled 48 h after the injection, with those of sham animals (surgery but no toxin injection). Data were Log transformed and refer to 6 treated versus 5 control animals (sham). Concentration data are Log transformed

In a recent paper (Basit et al. 2016) we described the strong upregulation of seven Sn2-polyunsaturated (20:4 or 22:6) N-acyl-phosphatidylethanolamines (NAPEs) occurring in the dorsal striatum of mice 48 h after unilateral injection of 6-OHDA in the striatum. This neurotoxic model for PD is very popular and useful to explore the molecular mechanisms of the disease (Bove et al. 2005). It has a series of advantages: the less extensive lesion (Przedborski et al. 1995) compared to other models, closer to PD, the ability to promote also non-motor symptoms and the likeliness of injection success (Sauer and Oertel 1994). Conversely, like other neurotoxic models, the response greatly depends on the injection success; this often generates high variability in the animal response to the toxin. Furthermore, the 6-OHDA model lacks the progressive and age-dependent effects of PD and it does not lead to the formation of Levy bodies (Tieu 2011). Bearing in mind all these cautions, we then tried to investigate if the NAPEs increase observed in the brain was accompanied by some alteration at peripheral level, and we sampled the plasma of the same animals at the same 48 h timepoint. We quantified the panel of seven NAPEs in plasma by using a slightly modified version of the method already described (Basit et al. 2016). The full description of the analytical method is reported in the Supplementary information. Rather unexpectedly, we observed a general trend for downregulation of these lipids in the 6-OHDA animals, with two NAPEs (16:0-22:6-N18:0 and 18:0-22:6-N16:0) being highly significantly downregulated in the plasma of 6-OHDA treated animals compared to the sham animals (surgery was done but no toxin was injected). The results are reported in Fig. 1.

Two out of the seven lipids show a significant decrease at the 0.05 level and one at the 0.15 level. The spread of the red points in the plot indicates that 6-OHDA treatment induced a stronger variability in the concentration of plasma lipids compared to control animals (sham, purple dots). The animals were sacrificed only 48 h after the injection and none of them was showing any PD symptoms, that normally occur 2 to 3 weeks after the insult (Przedborski et al. 1995). The results obtained for brain and plasma might suggest that 6-OHDA treatment alters the systemic levels of NAPEs and the increase in brain concentration is accompanied by a decrease in peripheral levels. We then decided to perform an exploratory study, by conducting a targeted metabolomics experiment. We measured the levels of these seven lipids in the plasma of a large cohort of subjects diagnosed with Parkinson’s disease and we compared them with the levels of a group of healthy subjects. No preliminary hypothesis was formulated for this experiment, as we wanted to explore any possible association between circulating NAPE levels and PD diagnosis. These subjects represent a generic population of PD patients or control subjects and no control on diet (other than overnight fasting before the blood withdrawal) was applied. It was not even possible to collect data on BMI values of these individuals or information on their lifestyle. Conversely, none of the PD patients, or their related caregiver, at the time of sampling reported any significant change in their usual diet following the diagnosis. The final patient distribution and sociodemographic data are reported in Table 1.

**Table 1:** Sociodemographic and clinical characteristics of males and females with Parkinson’s disease and healthy control subjects

	Control subjects	Parkinsons’s disease
Female	177	117
Male	142	151
Age (average-span)	52 (19–90)	63 (35–86)
Education	14.2 (5–27)	11.2 (5–25)
MMSE score	29.3 (26–30)	28.6 (26–30)

Supplementary Datafile 1 reports the full set of raw data from this human study; the concentration values were again Log transformed. As shown in Supplementary Fig. 1, the distribution of healthy controls spans over a broader range compared to PD patients. To correct for this, a different set of healthy controls (Age > 40) was considered during the statistical analysis, thus age matching the two groups. Supplementary Datafile 2 reports the final set of data used for the analysis after the rebalancing of the sample set and the batch effect removal. Table 2 reports the dataset composition after age matching.

**Table 2:** Distribution of male and female subjects in the final dataset used for the study

	Total	Control subjects	Parkinsons’s disease
Female	256	142	114
Male	263	114	149

**Fig. 2:**
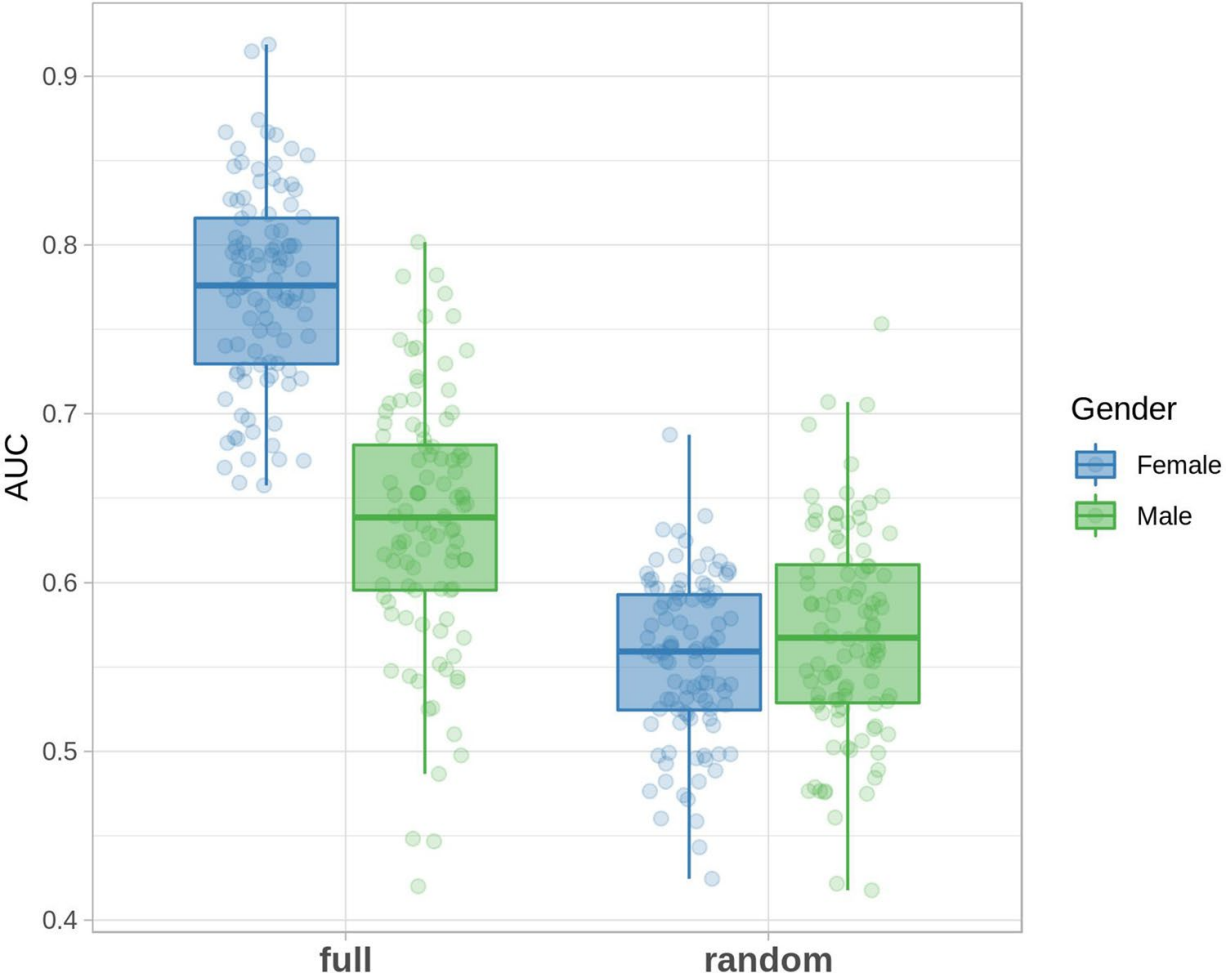
AUC values calculated for the selected panel of NAPEs compared to a random classifier for both male (green dots) and female (blue dots) subjects. Each point corresponds to the AUC calculated for an independent training-test split

The predictive potential of the proposed NAPE panel for the PD diagnosis was evaluated by Random Forest. In a recent systematic review and meta-analysis that investigated gender dependence in PD incidence (Hirsch et al. 2016), it has been demonstrated that while male subjects have a higher incidence of PD in all age groups, this difference is only statistically significant for women in age ranges 60–69 and 70–79. A potential explanation for the increased incidence in males not causing a parallel increase in prevalence is the more benign course of the disease in females, speculated to be caused by higher estrogen activity, which leads to higher dopamine levels in the striatum (Hirsch et al. 2016). Based on this evidence, and thus considering that the general risk of developing PD increases with age, we also added the subject’s age as a further variable to the RF model. As the original dataset was not well balanced for gender (F/M ratio: 1.2 for controls and 0.8 for PD), we performed two separated, independent analyses for female and male subjects. The corresponding AUC values were calculated for both genders, as reported in Fig. 2.

**Fig. 3:**
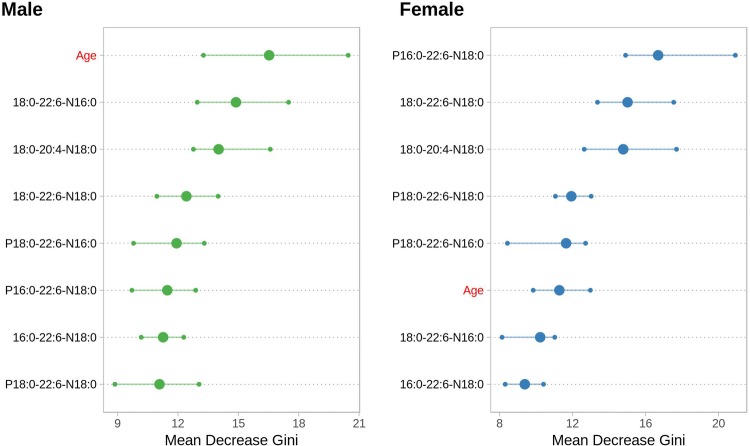
Variable Importance plots for the predictive model based on the plasma concentration of the seven NAPEs and the “age” factor. The importance of each variable is assessed in terms of Mean Decrease of Gini impurity index (see Sect. 2 for details). The horizontal segments indicate the 95 confidence intervals for each variable importance over the independent 100 models

The corresponding ROC curves at 95% confidence interval were also calculated and are reported in Supplementary Fig. 2. Our data indicate that the proposed panel of seven NAPEs and the variable “age” can be used to predict the patient status better than a random classifier for both genders. While for males the performance compared to the random classifier is quite low, the predicting power of NAPEs for PD in female subjects appears to be much stronger, thus suggesting that the panel of NAPEs is able to distinguish PD in women with higher specificity than in men. We then explored the importance of the individual variables on the overall model performance (see Sect. 2). Figure 3 reports the corresponding Variable Importance Plot for our classification model.

**Fig. 4:**
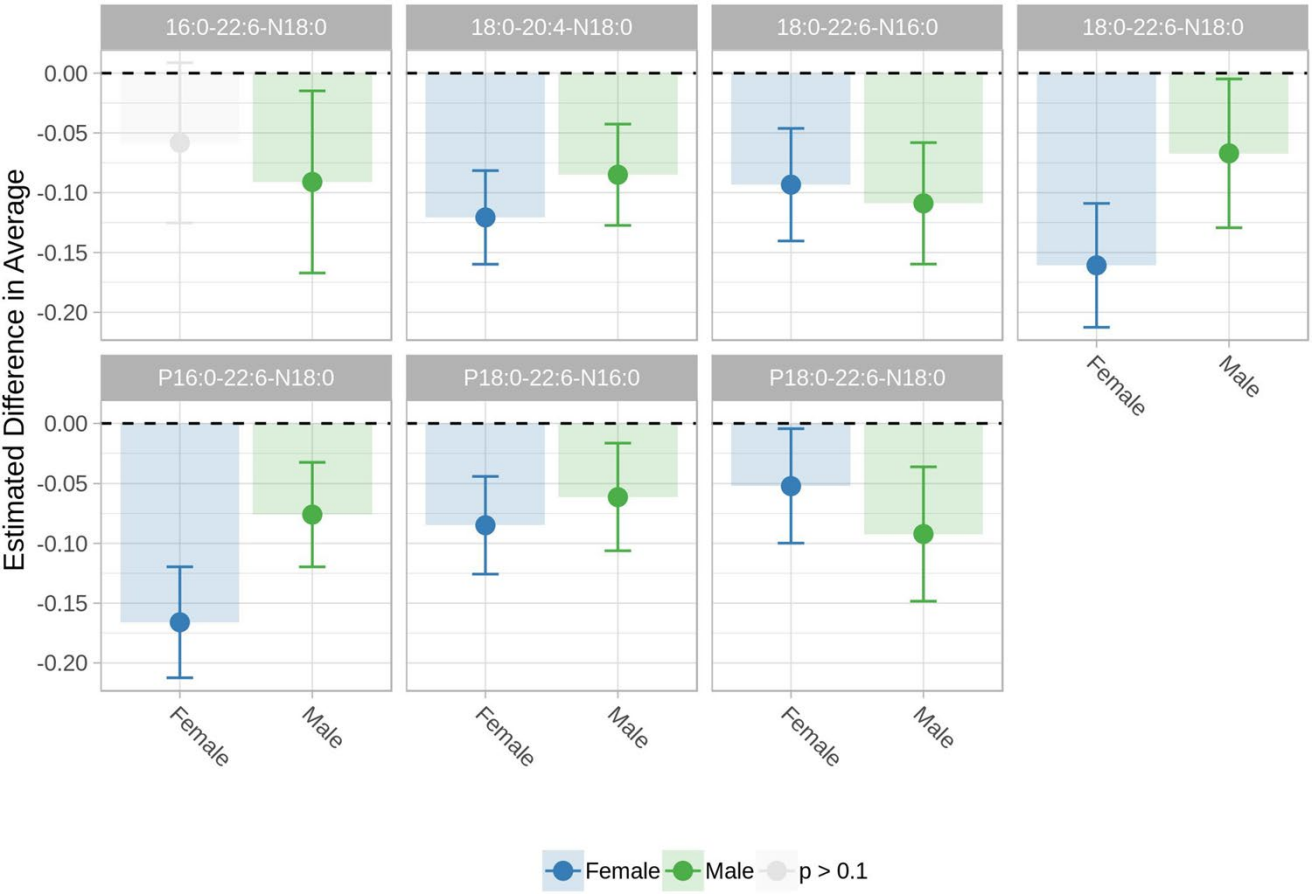
Gender-dependent downregulation of each NAPE in PD. The plot shows the average plasma concentration of each lipid (corrected for age) in males and females PD compared to controls subjects (whose average value is set to zero, dotted line). All the trends are significant (p < 0.1 after FDR correction) except for: 16:0-22:6-N18:0, not significant for Female subjects

In the plot, the importance of each variable is evaluated in terms of mean decrease in Gini Index (Wehrens 2011), highlighting the median value and the 95% confidence intervals in the 100 independent training/test splits. The full description of the model validation is reported in the Methods section. As clearly indicated by the plot, for male subjects, age (in red) is the most import variable for the model, while in female subjects, the importance of age in the model is limited and much lower than that of at least three circulating NAPEs (P16:0/22:6/N18:0), (18:0/22:6/N18:0), (18:0/20:4/N18:0). Both plots, show a comparable effects of the variables included in the model, thus suggesting that the combination of Age and NAPEs can be effective in predicting the disease status. It should be noted here that BMI might represent a potential confounding factor in our study, as NAPEs are mostly introduced with or produced from diet. Several papers show that BMI increases with age (Thevenot et al. 2015) and that, on average, it is higher in males that in females (Krumsiek et al. 2015; Trabado et al. 2017). Conversely, recent studies suggest that high BMI and obesity are not associated with Parkinson’s (Roos et al. 2018) and that higher BMI might be potentially protective toward PD (Noyce et al. 2017). We can then conclude that there is no reported evidence for higher BMI values in PD population and thus our conclusion on the potential of NAPEs for PD diagnosis in female patients still holds true. While our classification analysis confirms that the panel of lipids is useful to predict the disease state, it is not giving any information on the effect of the disease on the concentration of the single lipids. In addition, the presence of ‘age’ among the most predictive variables, suggests the possible presence of an age dependent effect on the concentration of the measured metabolites. In order to take into consideration this effect and investigate the effect of the disease on the concentration of the circulating lipids, the NAPE concentrations for male and female subjects were analyzed by using a linear model approach (Kreidler et al. 2013), explicitly including age as predictor in the model. Male and female subjects were analyzed *independently*, to account for the gender imbalance in the dataset. The results of the modeling of the seven lipids are presented in Fig. 4, which shows the average concentration of each lipid (corrected for age trends) in PD patients compared to the average concentration in healthy controls (set to zero).

Only parameters showing a p value lower than 0.1 after FDR correction were considered significant. Six out of seven lipids are significantly lower in *both* male and female PD subjects. NAPE 16:0-22:6-N18:0, is significantly decreased in PD compared with controls for male subjects only. The plots indicate that, for every lipid, the average concentration is lower in the disease state than in the controls, similarly to what we observed in the 6-OHDA mouse model of PD. From a translational perspective, the two lipids that are significantly downregulated in *male* mice (16:0-22:6-N18:0 and 18:0-22:6-N16:0) are also significantly downregulated in the plasma of *male* PD patients.

### A putative mechanism

NAPEs are precursors of lipid messengers but also important signaling molecules per se (Coulon et al. 2012). They are naturally ubiquitous in mammalian tissues (Petersen et al. 2005). Intriguingly, NAPEs have been shown to accumulate in tissues where degeneration is ongoing (Hansen et al. 1997, 2001). Based on our observation in mouse brain, we can thus speculate that their levels are higher also in PD human brains. Conversely, we now know that their levels are generally lower in PD patients’ plasma, and this is in line with our observations in 6-OHDA *mouse* plasma. We thus believe that central neurodegeneration causes a structural damage to neurons that is counterbalanced by an increased recruitment of NAPEs from blood. The aim of this redistribution would be to maintain cell membrane integrity and counteract cell death: indeed, several authors report that NAPEs, given their peculiar structure, play a crucial role in maintaining the biophysical integrity of biological membranes, especially in the central nervous system (Hansen et al. 1997). As a further supporting evidence to our hypothesis, it has already been demonstrated by using radiolabeled compounds that NAPEs deriving from food are actively recruited from the gut into the brain (Gillum et al. 2008) passing through the blood–brain barrier. It is also known that these lipids are synthesized in the small intestine (Gillum et al. 2008) also with the active participation of gut microbiota (Fu et al. 2007). Furthermore, recent data suggest that gut microbiota is crucial in neurodegeneration (Erny and Prinz 2017; Jiang et al. 2017; Mancuso and Santangelo 2017). This evidence itself might represent a link between our observations on NAPEs and the demonstrated role of gut microbiota on neurodegeneration. Our proposed scenario is that the altered gut microbiota of PD patients is not able to fully restore blood levels of NAPEs and thus is only partially able to contribute to the rebalancing of brain NAPEs. Nothing is known about the kinetics of this periphery/brain rebalancing and this deserves further investigations. The early stage effect that we observe in mice might be very different from phenomena occurring in patients, where the brain damage is progressive and associated to aging.

## Conclusions

Based on present findings, we can conclude that the downregulation of particular circulating NAPE species might have the potential to become a candidate biomarker for PD in female subjects. Our study shows, indeed, that NAPEs are significantly downregulated in the plasma of PD patients, with gender-specific profiles. To the best of our knowledge, this observation has never been reported before. We are also hereby proposing a simple molecular mechanism that might explain our findings. Transfer of NAPEs from circulating plasma to CNS appears to be not only plausible, but it has been already reported by others (Gillum et al. 2008). In the perspective of a future use of these lipids as biomarkers for PD, it could be argued that the average AUC values obtained with the ROC analysis in female subjects (0.75–0.85), are not sufficient to propose a highly specific analytical test potentially interesting in a clinical context. Furthermore, as discussed above, the absence of BMI data might represent a potential confounder in our work. However, considering that NAPEs are exclusively introduced or synthesized from food intake, it is remarkable that a clear effect is visible in a cohort of patients not stratified for BMI, diet and lifestyle. We thus believe that the present explorative work has produced ground for further extensive cross-validation works, with a more stringent control of patient stratification, also based on diet-related data like BMI. This is required to confirm the potential of NAPE lipids as candidate biomarkers for PD. We also believe that plasma NAPEs, being a relatively simple readout, might represent a valuable tool for pharmacological research and PD, if proven to be associated to disease progression and recovery. Furthermore, the link between NAPEs, diet and gut microbiome envisages a possible role of nutrition and bacterial engineering (Dosoky et al. 2017) as potential therapeutic opportunities to act on circulating NAPE levels and possibly restore or contrast CNS degeneration.

## Electronic supplementary material

Below is the link to the electronic supplementary material.

Supplementary material 1 (XLSX 90 kb)

Supplementary material 2 (XLSX 89 kb)

Supplementary material 3 (DOCX 137 kb)

## Data Availability

The full set of RAW data from our targeted analysis is fully available as Supplementary Datafile 1 (RAW Dataset).
