# Supplementary material for: Gender specific decrease of a set of circulating N-acylphosphatidyl ethanolamines (NAPEs) in the plasma of Parkinson’s disease patients
Source: Metabolomics. 2019 May 3;15(5):74. doi: 10.1007/s11306-019-1536-z (PMC6499742; doi:10.1007/s11306-019-1536-z)
Supplement: Supplementary file 3 — Supplementary material 3 (DOCX 137 kb) [file 11306_2019_1536_MOESM3_ESM.docx]

**SUPPLEMENTARY FIGURES**


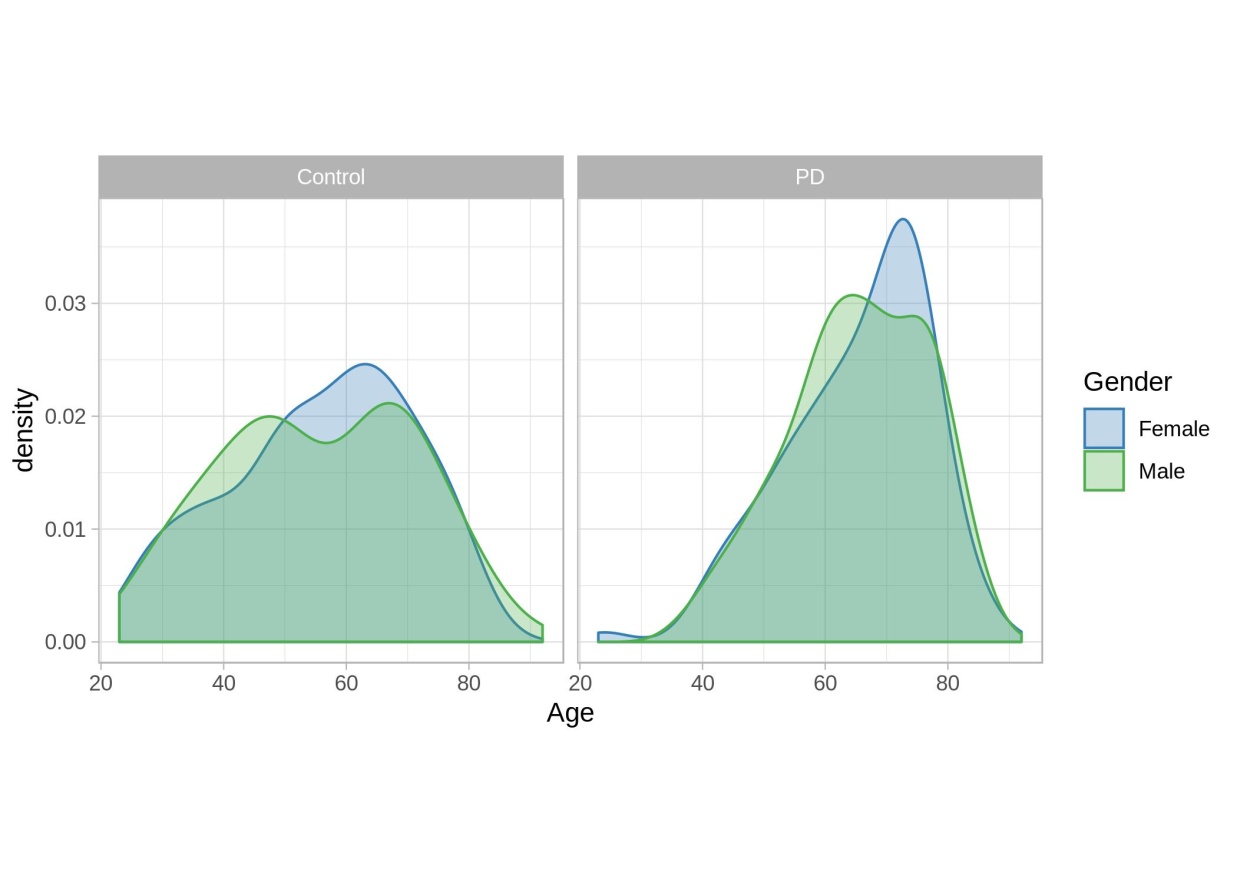


**Supplementary Figure 1**. *Subjects distribution by age.*


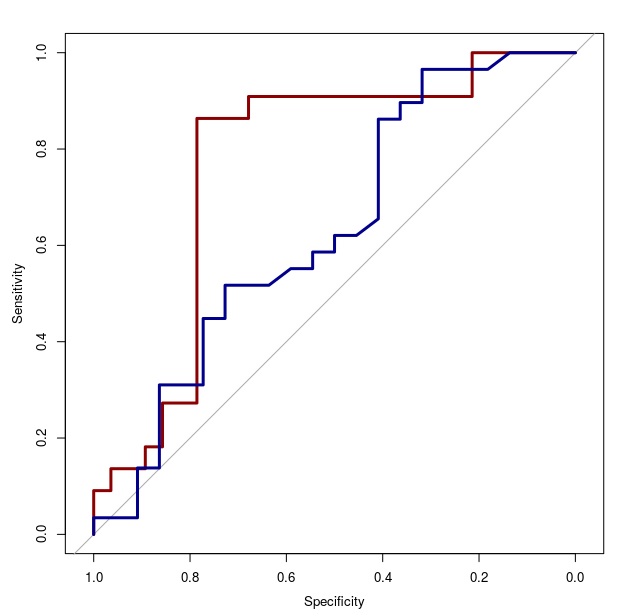


**Supplementary Figure 2.** *ROC values calculated at 95% confidence interval for both male (blue lines) and female (red line) subjects.*
